# Sedative and Anxiolytic Activities of *Opuntia ficus indica* (L.) Mill.: An Experimental Assessment in Mice

**DOI:** 10.3390/molecules25081844

**Published:** 2020-04-16

**Authors:** Esra Küpeli Akkol, Mert Ilhan, Büşra Karpuz, Yasin Genç, Eduardo Sobarzo-Sánchez

**Affiliations:** 1 Department of Pharmacognosy, Faculty of Pharmacy, Gazi University, Etiler, Ankara 06330, Turkey; busrakarpuz13@gmail.com; 2 Department of Pharmacognosy, Faculty of Pharmacy, Van Yüzüncü Yıl University, Tuşba 65080, Van, Turkey; mertilhan@yyu.edu.tr; 3 Department of Pharmacognosy, Faculty of Pharmacy, Hacettepe University, Sıhhiye, Ankara 06100, Turkey; yasin.genc@hacettepe.edu.tr; 4 Instituto de Investigación e Innovación en Salud, Facultad de Ciencias de la Salud, Universidad Central de Chile, 8330507 Santiago, Chile; 5 Department of Organic Chemistry, Faculty of Pharmacy, University of Santiago de Compostela, 15782 Santiago de Compostela, Spain

**Keywords:** anxiolytic, cactaceae, hypnotic, mice, *Opuntia ficus indica*, sedative

## Abstract

Ethnobotanical field surveys revealed the use of fruits of *Opuntia ficus indica* (L.) Mill. for treating diabetes, burns, bronchial asthma, constipation, kidney stones, and rheumatic pains and as a sedative in Turkish folk medicine. This study aimed to verify the efficacy of the fruits of *O. ficus indica* experimentally and to define components responsible for the activity using bioassay-guided procedures. The crude methanolic extract of the fruits was sequentially fractionated into five subextracts: *n*-hexane, dichloromethane, ethyl acetate, *n*-butanol, and water. Further experiments were carried out on the most active subextract, that is, the ethyl acetate (EtOAc) subextract, which was further subjected to fractionation through successive column chromatographic applications on Sephadex LH-20. For activity assessment, each extract or fraction was submitted to bioassay systems; traction test, fireplace test, hole-board test, elevated plus-maze test, and open-field test were used for sedative and anxiolytic effects, and a thiopental-induced sleeping test was used for the hypnotic effect. Among the subextracts obtained from the methanolic extract, the EtOAc subextract showed significant sedative and anxiolytic effects in the bioassay systems. From the EtOAc subextract, major components were isolated, and their structures were determined as isorhamnetin, isorhamnetin 3-*O*-glucoside, isorhamnetin 3-*O*-rutinoside, and kaempferol 3-*O*-rutinoside using spectral techniques. In conclusion, this study confirmed the claimed use of the plant against anxiety in Turkish folk medicine.

## 1. Introduction

Common anxiety disorder is the most common among mental disorders in patients of advanced age group [1]. In general, anxiety disorders can be treated with certain psychotherapeutic drugs. Sedatives and hypnotics are medications that can reduce anxiety and induce the onset of sleep and maintain sleep time [2]. Benzodiazepines are commonly used because of their muscle-relaxant, sedative-hypnotic, and anticonvulsant effects [3]. However, the continued use of these currently available sedative-hypnotic treatments has serious side effects, from respiratory, digestive, and immune system dysfunctions to impaired cognitive function, physical dependence, and tolerance [4]. Therefore, the development of new sedative-hypnotic drugs with fewer side effects has been suggested as a promising approach to combat different psychiatric disorders.

*Opuntia ficus indica* (L.) Miller, known as “Hint inciri” in Turkey, is a cactus species that can reach a height up to 5 m. It belongs to the Cactaceae family [5,6]. *O. ficus indica*, one of the few *Opuntia* species that produces edible fruit, is of Mexican origin and is found in Sicily [7,8,9], the Mediterranean Basin [10], arid plateaus of western Asia [11], and the south-western USA. It is of economic importance in terms of agriculture [12,13,14]. It is used in many regions to control wind and water erosion [13,15], and in dry regions to control soil erosion. It is also used as a feed substitute during drought [11]. *Opuntia* species are a natural, rich source of dietary fiber. Fruits of the cactus are usually consumed as food after peeling them when they are fresh. Moreover, they are used in the preparation of fruit juice, jelly, jam, sugar, coloring food, ice cream, and other foods, and also in cosmetics [16,17,18]. It is used for treating diabetes, burns, bronchial asthma, and indigestion in many countries in the world due to the strong antioxidant effects of its fruits [19]. *O. ficus indica* fruits are used as a laxative in Turkey to reduce kidney stones, rheumatism pains, and as a sedative [20].

Previous phytochemical studies performed on *O. ficus indica* revealed the presence of pigments, betalains [21], polyphenols, vitamins C and E, minerals (potassium, phosphorus, magnesium, sodium, and calcium) [22,23], glutamine, proline, taurine [24,25,26,27,28], quercetin glycosides, isorhamnetin 3-*O*-rutinoside, and isorhamnetin triglycosides [29]. Fruits and skin are particularly rich in betacyanins and betaxanthins [30,31]. Color pigments are also present in different amounts and types of fruits [32,33].

Investigations on the biological activity revealed the diuretic, antigotous [34], anti-inflammatory, analgesic [35,36], antiulcer [32,37,38], neuroprotective, antihyperglycemic, and hypocholesterolemic effects of *O. ficus indica* [39,40].

The aim of the present study was to evaluate the sedative activity of the extracts from the fruits of *O. ficus indica*, isolate and define the active constituent(s) using bioassay-guided fractionation procedures, and find out the activity mechanisms.

## 2. Results and Discussion

This study investigated the sedative and anxiolytic effects of the extracts prepared from the fruits of *O. ficus indica*. The traction test, fireplace test, hole-board test, elevated plus maze (EPM) test, and open-field test were used for evaluating sedative and anxiolytic effects, while the thiopental-induced sleeping test was used for evaluating the hypnotic effect.

**Figure 1:**
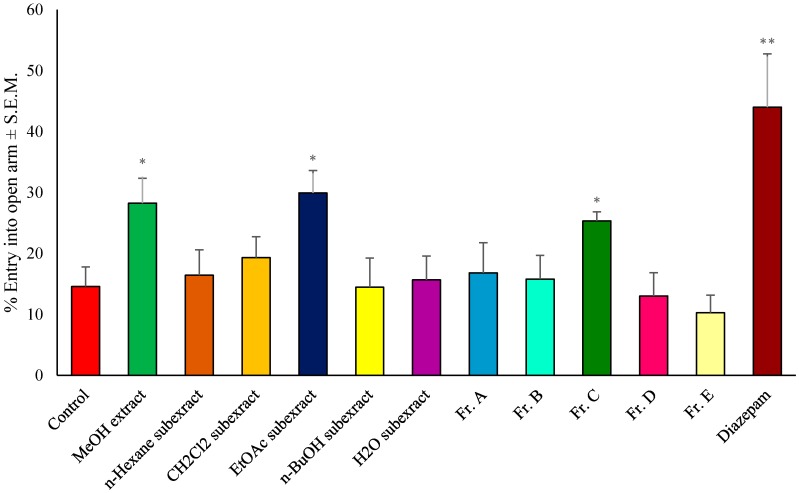
Effects of the test materials on elevated plus maze test. * *p* < 0.05; ** *p* < 0.01; S.E.M.: Standard Error of Mean.

In the preliminary activity assessment, the MeOH extract prepared from the fruits was tested in the bioassay systems. Among the subextracts obtained through successive solvent extractions, the EtOAc subextract was found to be the most active in experimental models (Table 1, Table 2 and Table 3, Figure 1).

Later, this subextract was fractionated by successive column chromatography techniques, and the fractions were submitted to the same bioassay systems. Fr. C was found to be the most active fraction (Table 1, Table 2 and Table 3). Successive bioassay-guided fractionation procedures yielded a major component from the active fraction. The structures of these components were elucidated to be isorhamnetin [41], isorhamnetin 3-*O*-glucoside [42], isorhamnetin 3-*O*-rutinoside [42], and kaempferol 3-*O*-rutinoside [42] by comparing the one- and two-dimensional NMR data with previously reported data.

Diazepam is a benzodiazepine that acts by maximizing pre- and postsynaptic inhibition of γ-aminobutyric acid (GABA) in various synapses through interacting with specific benzodiazepine receptors in the central nervous system (CNS) [43]. The inhibition of 5-hydroxytryptophan (5-HT) and noradrenergic neurons may be responsible for anxiolytic and sedative effects [43]. For this purpose, diazepam was used as the reference standard in the present study.

The hole-board test is a very important and preferred method for investigating the potential sedative and anxiolytic effects of any agent in rodents. This test is advantageous due to its methodological simplicity. Also, it can easily display various behavioral responses if the experimental animal is exposed to a foreign body or condition. The head-dipping behavior is directly related to the emotional state of animals [44]. There is a correlation with the increase in head dipping behavior in animals [44] head dipping number [45]. As seen in Table 1, after the applications of the MeOH extract, the EtOAc subextract, Fr. C, and diazepam in the hole-board test, the number of head insertion decreased statistically significantly compared with the head insertion in the control group. The other extracts and fractions did not cause a significant change in the number of head insertion into the hole. The results showed that *O. ficus indica* had sedative and anxiolytic effects.

Traction test is a frequently preferred method for determining muscle-relaxant and sedative activities [46]. In this study, the muscle-relaxant and sedative activities of extracts and fractions prepared from *O. ficus indica* fruits were compared with those of the reference drug diazepam using the traction test. However, in this study, none of the extracts and fractions showed any muscle-relaxant activity compared with the control group (Table 1).

In the fireplace test, the mice were treated with extracts and fractions from *O. ficus indica*. A significant difference was found in the escape time compared with the control group. The MeOH extract, the EtOAc subextract, Fr. C, and diazepam groups presented relatively longer escape time compared with the other test groups (Table 1).

The EPM test was used to test the behavioral, physiological, and pharmacological effects of drugs by testing emotional activity [47,48,49,50]. This experimental setup, which had two open and two closed arms at a certain height from the ground, was used to evaluate the time taken by the mouse placed in the closed arm to enter the open arm and the length of stay in this arm. Studies have shown that anxiolytic agents increase while anxiogenic agents decrease this time [51,52,53].

In the present study, the MeOH extract, the EtOAc subextract, and Fr. C were found to increase the duration of stay in open arms, using diazepam as a reference drug. Therefore, these extracts and fractions were thought to act on GABA receptors such as diazepam (Figure 1).

Anxiety behavior in the animal kept in an open area is triggered by two factors: animal left alone in an unfamiliar environment and the fear of wide area called agoraphobia [54]. The open-field test performed for this purpose is one of the most commonly used tests to determine the emotional state of experimental animals before the procedure and any changes that may occur after the procedure [55]. It is also a test used to detect emotions, locomotor activity, and sedation that develop due to anxiety [56]. Locomotor activity is an indicator of mental wakefulness or alertness. Decreased locomotion, which is indicative of calmness and sedation, can be interpreted as reduced CNS excitability [57]. In the present study, the MeOH extract, the EtOAc subextract, and Fr. C obtained from the EtOAc subextract exerted an anxiolytic effect by affecting locomotor activity 30 min after the start of the experiment (Table 2).

The inhibition of GABA involves opening of chloride channels that allow hyperpolarization of the membrane, lead to CNS depression, and cause sedative and hypnotic activities. Glutamate and GABA are the most important stimulating and inhibitory neurotransmitters in the mammalian brain. Therefore, these two neurotransmitter receptors are considered important targets for psychotropic drugs. Thiopental, a member of the barbiturate group, can trigger sleep in both humans and rodents. The thiopental-induced sleeping time test is one of the most preferred experiments in the study of sedative-hypnotic drugs [43]. CNS depressant barbiturates, such as thiopental sodium, bind to the barbiturate-binding site on the GABA receptor complex and induce GABA-mediated hyperpolarization of postsynaptic neurons [43]. In the present study, the MeOH extract, the EtOAc subextract, and Fr. C obtained from the EtOAc subextract were found to increase sleep time compared with the control group (Table 3). Considering that the inhibitory effect of thiopental on the CNS is associated with activation of the GABAergic system [58,59], the results of the present study showed that the sedative-hypnotic effect of some components of *O. ficus indica* was based on GABAergic receptors.

Some previous studies showed that plants containing alkaloids, flavonoids, terpenes, and saponins had sedative, anxiolytic, and antiepileptic properties due to the affinity of the GABAergic system for the benzodiazepine region [60,61,62,63,64]. Many plants with a depressant effect due to flavonoids affecting central benzodiazepine receptors on the CNS are used in folk medicine [65]. Flavonoids display anxiolytic, sedative, anticonvulsant, and analgesic properties by interacting with various receptors and signaling systems, including GABA receptors [66,67,68,69]. They have been shown to modulate GABAA receptors at low concentrations with or without sensitivity to flumazenil [70]. Therefore, they can affect GABAergic system receptors through the classical benzodiazepine-binding site and also independently of this site. Previous studies showed that apigenin combined with diazepam strongly modulated IGABA (GABA-induced chloride current) [45,71,72]. Rutin is another abundant flavonoid detected in plants. Further, plants increase glycosylated aglycone production as a mechanism to avoid damage [73]. This property of plants is also an advantage in folk medicine because these plants can be used as tranquilizers. It was suggested that the flavonoids in *O. ficus indica* contributed to its hypnotic effect via central benzodiazepine receptors.

Oxidation and associated inflammation are important factors in the development of neurodegenerative diseases. Therefore, the use of antioxidant and anti-inflammatory compounds is highly advantageous in neurodegenerative disorders. Dok-Go et al. reported a neuroprotective effect of *O. ficus indica* in primary cultured rat cortical cells [74]. Quercetin, (+)-dihydroquercetin, and quercetin 3-methyl ether, which has the flavonoid structure in the composition of *O. ficus indica*, were shown to have antioxidant and neuroprotective effects [74]. In addition, quercetin was reported to have a neuroprotective effect against *N*-methyl-d-aspartate (NMDA), kainate (KA), and oxygen-glucose deprivation (OGD)-caused neurotoxicity in cultured rat cortical cells [75]. Clarke and Ramsay reported that isorhamnetin showed the remarkable monoamine oxidase A inhibitory activity [76]. On the other hand, Asha and Sumathi revealed that isorhamnetin displayed potent neuroprotective effect against Amyloid beta induced neurotoxicity in rats [77]. Kim et al. demonstrated a neuroprotective effect of *O. ficus indica* methanol extract against NMDA-, KA-, and OGD-caused neuronal damage in mouse cortical cell culture [19]. *O. ficus indica* exerts anti-inflammatory and neuroprotective effects due to a component called nicotiflorin. It decreases the damaged tissue size in the brain infarct and reduces the neurological damage due to the increase in the level of ischemia and endothelial nitric oxide synthase [78]. Nakayama et al. reported that nicotiflorin was found to be neuroprotective against retinal ganglion cell death induced by hypoxia, glutamate, and oxidative stress even at nanomolar concentrations [79]. In the Morris water maze test in mice, nicotiflorin showed a protective effect against memory oxidative stress by preventing the increase in lactic acid and malondialdehyde levels [80].

## 3. Materials and Methods

### 3.1. Plant Material

Fruits of the plant were collected from the garden of Palm Villas in Belek, Antalya, Turkey, in August 2018 and identified by Prof. Dr. Esra Küpeli Akkol from Gazi University, Faculty of Pharmacy, Ankara, Turkey. A voucher specimen (GUEF2315) has been kept in the Herbarium of the Faculty of Pharmacy, Gazi University.

### 3.2. Extraction and Fractionation Processes for the Bioassays

*O. ficus indica* fruits were rinsed, dried at room temperature for 1 day, and then chopped into little pieces. Then, 500 g fresh material was extracted with methanol at room temperature for 3 days (3 × 1 L). The combined methanol extract was evaporated to dryness in vacuo to yield the methanol (MeOH) extract (28.91%).

The residual extract was fractionated by successive solvent extractions with *n*-hexane (4 × 1 L), dichloromethane (CH_2_Cl_2_) (4 × 1 L), ethyl acetate (EtOAc) (4 × 1L), *n*-butanol (*n*-BuOH) (4 × 1 L), and H_2_O (4 × 1 L). Each subextract in addition to the remaining aqueous subextract after solvent extractions was vaporized to dryness under reduced pressure to yield “*n*-hexane subextract” (2.3%), “CH_2_Cl_2_ subextract” (14.7%), “EtOAc subextract” (37.8%), “*n*-BuOH subextract” (7.8%), and “H_2_O subextract” (10.1%).

### 3.3. Chromatographic Separation and Isolation of the Active Compounds

The EtOAc subextract was fractionated with MeOH on the Sephadex LH-20 column to yield four main fractions: Frs. A–E (Fr. A, 6.1 g; Fr. B, 10.3 g; Fr. C, 14.6 g; Fr. D, 10.2 g; Fr. E, 5.9 g). Fr. C, the most active fraction, was further subjected to chromatographic separation on the Silica column using 2 L of EtOAc:CHCl_3_:MeOH:H_2_O (15:8:4:1) and 2 L EtOAc:CHCl_3_:MeOH:H_2_O (6:4:4:1) as eluents. Compound 1 (100.3 mg), compound 2 (65.4 mg), compound 3 (34.8 mg), and compound 4 (56.7 mg) were isolated in pure form.

### 3.4. Structure Elucidation of the Compounds 1–4

**Figure 2:**
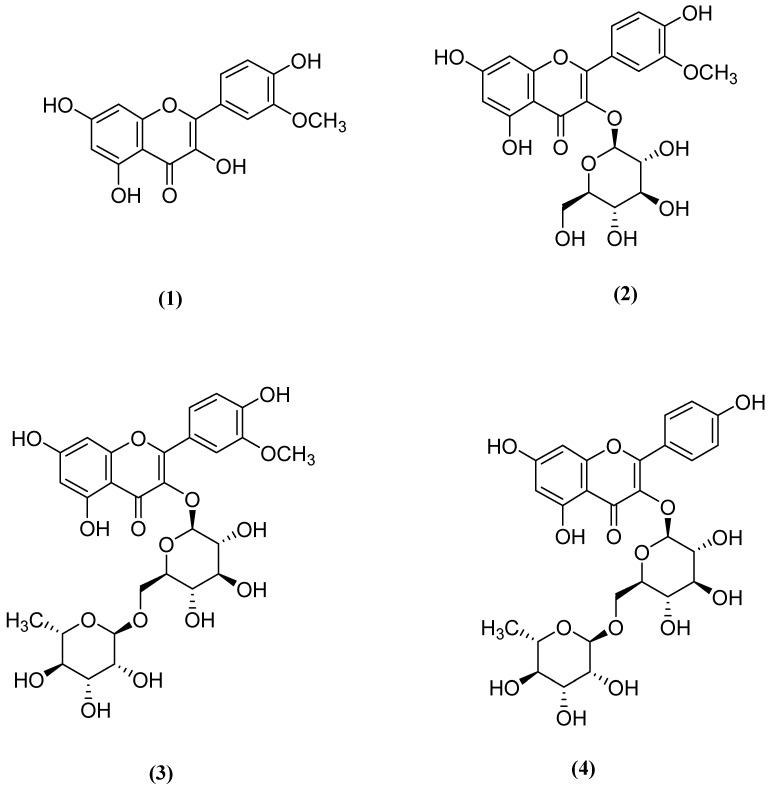
Chemical structures of isorhamnetin (**1**), isorhamnetin 3-*O*-glucoside (**2**), isorhamnetin 3-*O*-rutinoside (**3**), and kaempferol 3-*O*-rutinoside (nicotiflorin) (**4**) isolated from *Opuntia ficus indica.*

The structures of the isolated compounds from *Fr. C* were determined by spectral techniques such as 1D- and 2D-NMR (^1^H-, ^13^C-NMR, Bruker^®^, San Jose, CA, USA) and mass spectroscopy (Waters LCT Premier XE UPLC/MS-TOF, Milford, MA, USA). The chemical names of compounds 1–4 were as follows (Figure 2): (1) isorhamnetin, (2) isorhamnetin 3-*O*-glucoside, (3) isorhamnetin 3-*O*-rutinoside, and (4) kaempferol 3-*O*-rutinoside (nicotiflorin). The NMR data of compounds 1, 2, 3, and 4 were given in Supplementary Materials.

### 3.5. Biological Activity Studies

#### 3.5.1. Animals

BALB/c male mice, weighing 25–30 g, were obtained from the Experimental Animal Production and Research Laboratory of Kobay Firm and used in the experiments (Ankara, Turkey). They were kept under laboratory conditions for at least 3 days to adapt to the environment before starting the experiments. During this waiting period, they were fed a standard pellet feed and water, and housed in the laboratory under the following conditions: temperature, 21–24 °C, humidity, 40–45%, and 12-h light and 12-h dark. Six animals were used in each group in the experiments. All the studies were performed conferring to the international rules regarding the animal experiments and biodiversity rights. The experiment was approved by the Experimental Animal Ethics Committee of Kobay (Kobay Ethical Council Project Number: 478).

#### 3.5.2. Preparation of Test Samples

In the biological activity test models, the test samples were suspended in 0.5% sodium carboxymethyl cellulose (CMC) solution with the help of an ultrasonic bath when necessary. They were administered to the experimental animals at a dose of 100 mg/kg intraperitoneally. The control group mice were given 0.5% CMC, which was used only in the preparation of test samples. Diazepam (Sigma-Aldrich, St. Louis, MO, USA, CAS No: 439-14-5), used as a test sample and reference material, was administered to mice intraperitoneally at a dose of 1 mg/kg.

#### 3.5.3. Traction Test

The method developed by Courvoisier, Laroche, and Rousselet was used for this test [81,82]. Diazepam, which was used as a test sample and reference material in mice, was administered intraperitoneally 1 h after the mice were hung from the forefoot of the horizontally stretched rope. The mouse that could pull its hind legs to reach the rope was considered normal, whereas a mouse that failed to pull at least one of its hind legs to reach the rope was considered sedative. In addition, the behavior of the animals was recorded during the duration of the experiment.

#### 3.5.4. Fireplace Test

The method developed by Hoffman was used for this test [83]. Diazepam, which was used as a test sample and reference material in mice, was placed in a 30-cm-long vertical standing glass tube 1 h after intraperitoneal administration. If the mouse placed in the tube attempted to escape within 30 s, it was considered normal, while the mouse that did not attempt any more after this period was considered sedative.

#### 3.5.5. Hole-Board Test

The method developed by File and Wardill was used for this test [84,85]. A mouse was placed in the middle of a device of 40 × 40 × 25 cm^3^ with a hole of 2.2 cm in diameter on the ground 1 h after the intraperitoneal administration of diazepam as a test sample and reference material. The number of animals’ heads inserted into the holes on the device was recorded. The lower explored holes during 5 min were considered sedative.

#### 3.5.6. Elevated Plus-Maze Test

The effect of the test samples on the performance of mice in the elevated plus-maze (EPM) test was determined by the method proposed by Emamghoreishi et al. with some modifications [86]. The maze apparatus consisting of two open arms (5 × 10 × 0.5 cm^3^) and two closed arms (5 × 10 × l5 cm^3^) was used. Arms that spread from a central platform (5 × 5 cm^2^) were raised to 40 cm above the ground. Each mouse was placed in the center of the platform for 5 min 1 h after the treatments. Then, the number of open arm entries (all of the four claws outstretched was defined as an open arm entry) was recorded. Diazepam (1 mg/kg intraperitoneal dose) was used as a reference drug in this test. The higher open arm entry was considered as sedative.

#### 3.5.7. Open-Field Test

This test was used to investigate spontaneous locomotive and exploration activity in mice. An open-field apparatus consisting of a white box of 30 × 50 cm^2^ and a 27-cm wall was used. First, the area of the open field was divided into square blocks and colored in black and white. The mice were placed in the open-field apparatus 1 h after the application of test samples, and the number of square blocks visited by each mouse was calculated for 3 min at intervals of 30, 60, 90, and 120 min. If the number of squares crossed were low, the rats were considered as sedative. Diazepam was administered intraperitoneally at a dose of 1 mg/kg as a reference material [87].

#### 3.5.8. Thiopental-Induced Sleeping Test

The method developed by Aziz and Khan was used for this test [88,89,90]. The test samples were administered intraperitoneally within 30 min after exposure to thiopental at 60 mg/kg dose. The time from application till sleep and the time from sleep till wake up were recorded.

### 3.6. Statistical Analysis of Data

“Instat” (Windows) statistics program including one-way analysis of variance and Students–Newman–Keuls post-hoc test were used to evaluate the experimental results for active samples. The results were compared with the control and reference groups. Statistical significance was described as follows: * *p* < 0.05, ** *p* < 0.01, *** *p* < 0.001.

## 4. Conclusions

The findings of this study showed that *O. ficus indica* had strong sedative and hypnotic activities. The study was novel in describing the sedative and anxiolytic effects of *O. ficus indica*. However, extensive studies are needed to evaluate the precise mechanism(s) and the safety profile of the plant as a remedy for CNS disorders.

## Figures and Tables

**Table 1:** Sedative effects of test materials determined by traction, fireplace and holeboard tests.

Test Material	Dose (mg/kg)	Traction Test	Fireplace Test	Hole Board Test
		(Re-Establishment Time) (Sec) ± S.E.M	(Time to Go Back the Tube in Seconds) ± S.E.M	(Explored Holes During 5 min) ± S.E.M
*Control*	-	0.25 ± 0.05	10.63 ± 2.68	56.22 ± 9.74
*MeOH extract*	100	4.52 ± 1.26	159.27 ± 9.94 ***	8.78 ± 1.52 **
*n-Hexane subexract*	100	0.37 ± 0.11	14.79 ± 3.35	47.30 ± 10.97
*CH_2_Cl_2_ subexract*	100	1.74 ± 0.99	19.04 ± 2.81	39.04 ± 7.58
*EtOAc subexract*	100	3.04 ± 1.13	146.90 ± 11.04 ***	7.51 ± 1.83 **
*n-BuOH subexract*	100	2.56 ± 0.82	28.15 ± 5.66	42.69 ± 6.16
*H_2_O subexract*	100	0.62 ± 0.14	8.71 ± 1.04	54.93 ± 10.39
*Fr. A*	100	2.55 ± 1.83	12.40 ± 1.33	38.85 ± 8.07
*Fr. B*	100	3.10 ± 1.57	14.85 ± 3.62	35.22 ± 7.04
*Fr. C*	100	4.30 ± 0.75	156.36 ± 8.41	7.90 ± 1.36 **
*Fr. D*	100	2.31 ± 1.19	13.99 ± 3.79	28.74 ± 13.66
*Fr. E*	100	1.98 ± 0.63	10.32 ± 2.86	48.64 ± 12.59
*Diazepam*	1	11.82 ± 1.54 ***	174.61 ± 6.46 ***	0.00 ± 0.00 ***

** *p* < 0.01; *** *p* < 0.001; S.E.M.: Standard Error of Mean. Bold values represent a significant difference.

**Table 2:** Effect of the test materials on open field test.

Test Material	Dose (mg/kg)	Number of Squares Crossed ± S.E.M.			
		30 min	60 min	90 min	120 min
*Control*	-	78.84 ± 10.06	61.29 ± 9.93	43.31 ± 8.78	50.67 ± 7.02
*MeOH extract*	100	33.16 ± 7.42 *	30.41 ± 7.18 *	24.11 ± 6.07 *	19.93 ± 4.35 **
*n-Hexane subexract*	100	85.29 ± 13.61	75.53 ± 11.12	51.80 ± 14.21	50.19 ± 12.69
*CH_2_Cl_2_subexract*	100	74.13 ± 9.89	66.90 ± 10.71	58.59 ± 8.66	48.26 ± 9.07
*EtOAc subexract*	100	31.73 ± 7.26 *	28.82 ± 6.19 *	21.63 ± 9.91 *	16.07 ± 7.74 *
*n-BuOH subexract*	100	74.90 ± 11.42	57.14 ± 9.43	40.30 ± 8.53	41.27 ± 9.05
*H_2_O subexract*	100	81.99 ± 13.36	66.72 ± 14.59	51.34 ± 10.26	48.15 ± 11.39
*Fr. A*	100	74.62 ± 8.94	55.19 ± 8.90	39.91 ± 10.69	38.19 ± 12.23
*Fr. B*	100	75.86 ± 11.70	63.44 ± 9.76	48.24 ± 7.61	49.78 ± 9.19
*Fr. C*	100	30.16 ± 6.91 *	26.39 ± 5.82 **	21.74 ± 5.12 **	19.63 ± 4.85 **
*Fr. D*	100	79.83 ± 15.43	75.16 ± 12.41	68.60 ± 11.29	64.12 ± 9.01
*Fr. E*	100	71.10 ± 10.28	63.49 ± 8.29	60.31 ± 9.06	55.26 ± 10.92
*Diazepam*	1	25.14 ± 7.99 ***	20.43 ± 7.34 ***	14.35 ± 6.42 ***	11.20 ± 3.87 ***

* *p* < 0.05; ** *p* < 0.01; *** *p* < 0.001; S.E.M.: Standard Error of Mean. Bold values represent a significant difference.

**Table 3:** Effect of the test materials on thiopental sodium-induced sleeping time.

Test Material	Dose (mg/kg)	Onset of Sleeping (min) ± S.E.M	Sleeping Duration (min) ± S.E.M
*Control*	-	60.22 ± 5.99	74.14 ± 9.01
*MeOH extract*	100	26.81 ± 2.65 **	255.62 ± 4.80 ***
*n-Hexane subexract*	100	56.47 ± 7.16	81.49 ± 9.14
*CH_2_Cl_2_subexract*	100	47.35 ± 4.68	96.63 ± 13.43
*EtOAc subexract*	100	29.33 ± 1.94 **	234.91 ± 5.62 ***
*n-BuOH subexract*	100	58.40 ± 9.39	76.28 ± 11.89
*H_2_O subexract*	100	62.11 ± 8.53	61.13 ± 9.91
*Fr. A*	100	55.83 ± 10.90	88.43 ± 8.24
*Fr. B*	100	48.29 ± 9.77	79.01 ± 10.40
*Fr. C*	100	23.24 ± 2.10 **	249.15 ± 6.47 ***
*Fr. D*	100	41.35 ± 4.86	92.18 ± 12.63
*Fr. E*	100	68.91 ± 11.53	76.51 ± 9.94
*Diazepam*	1	12.04 ± 1.88 ***	310.53 ± 8.62 ***

** *p* < 0.01; *** *p* < 0.001; S.E.M.: Standard Error of Mean. Bold values represent a significant difference.

## Supplementary Materials

The following are available online, ^1^H and ^13^C-NMR data of compounds **1**, **2**, **3**, and **4**.

## References

[1] Lieb R., Becker E., Altamura C. (2005). The epidemiology of generalized anxiety disorder in Europe. Eur. Neuropsychopharmacol..

[2] Katzung B.G., Masters S.B., Trevor A.J. (2009). Basic and Clinical Pharmacology.

[3] Woods J.H. (1992). Benzodiazepines: Use, abuse, and consequences. Pharmacol. Rev..

[4] Dhawan K., Dhawan S., Chhabra S. (2003). Attenuation of benzodiazepine dependence in mice by a tri-substituted benzoflavone moiety of *Passiflora incarnata* Linneaus: A non-habit forming anxiolytic. J. Pharm. Pharm. Sci..

[5] Duru B., Turker N. (2005). Changes in physical properties and chemical composition of cactus pear (*Opuntia ficus-indica*) during maturation. J. Prof. Assoc. Cactus.

[6] Halmi S., Benlakssira B., Bechtarzi K., Djerrou Z., Djeaalab H., Riachi F., Pacha Y.H. (2012). Antihyperglycemic activity of prickly pear (*Opuntia ficus-indica*) aqueous extract. IJMAP.

[7] Galati E.M., Monforte M.T., Tripodo M.M., d’Aquino A., Mondello M.R. (2001). Antiulcer activity of *Opuntia ficus indica* (L.) Mill. (Cactaceae): Ultrastructural study. J. Ethnopharmacol..

[8] Rodriguez-Felix A. (2000). Postharvest physiology and technology of cactus pear fruits and cactus leaves. Acta Hortic..

[9] Russell C.E., Felker P. (1987). The prickly-pears (*Opuntia* spp., Cactaceae)—A source of human and animal food in semiarid regions. Econ. Bot..

[10] Le Houérou H.N. (2002). Cacti (*Opuntia* spp.) as a fodder crop for marginal lands in the Mediterranean basin. Acta Hortic..

[11] Le Houérou H.N. (2000). Utilization of fodder trees and shrubs in the arid and semiarid zones of West Asia and North Africa. Arid Soil Res. Rehab..

[12] Gregory R.A., Felker P. (1992). Crude protein and phosphorus contents of eight contrasting *Opuntia* forage clones. J. Arid Environ..

[13] Guevara J.C., Yahia E.M., De La Fuente E.B. (2001). Modified atmosphere packaging of prickly pear cactus stems (*Opuntia* spp.). LWT-Food Sci. Technol..

[14] Parish J., Felker P. (1997). Fruit quality and production of cactus pear (*Opuntia* spp.) fruit clones selected for increased frost hardiness. J. Arid Environ..

[15] Yahia E.M., Mondragon-Jacobo C. (2011). Nutr.itional components and anti-oxidant capacity of ten cultivars and lines of cactus pear fruit (*Opuntia* spp.). Food Res. Int..

[16] El-Samahy S.K., El-Hady E.A., Habiba R.A., Moussa-Ayoub T.E. (2007). Some functional, chemical, and sensory characteristics of cactus pear rice-based extrudates. J. Prof. Assoc. Cactus.

[17] El-Samahy S.K., Youssef K.M., Moussa-Ayoub T.E. (2009). Producing ice cream with concentrated cactus pear pulp: A preliminary study. J. Prof. Assoc. Cactus.

[18] Moßhammer M.R., Stintzing F.C., Carle R. (2006). Cactus pear fruits (*Opuntia* spp.): A review of processing technologies and current uses. J. Prof. Assoc. Cactus.

[19] Kim J.H., Park S.M., Ha H.J., Moon C.J., Shin T.K., Kim J.M., Lee N.H., Kim H.C., Jang K.J., Wie M.B. (2006). *Opuntia ficus-indica* attenuates neuronal injury in in vitro and in vivo models of cerebral ischemia. J. Ethnopharmacol..

[20] Gurdal B., Kultur S. (2013). An ethnobotanical study of medicinal plants in Marmaris (Mugla, Turkey). J. Ethnopharmacol..

[21] Osorio-Esquivel O., Alicia-Ortiz-Moreno, Alvarez V.B., Dorantes-Alvarez L., Giusti M.M. (2011). Phenolics, betacyanins and antioxidant activity in *Opuntia joconostle* fruits. Food Res. Int..

[22] El Kossori R.L., Villaume C., El Boustani E., Sauvaire Y., Mejean L. (1998). Composition of pulp, skin and seeds of prickly pears fruit (*Opuntia ficus indica* sp.). Plant Foods Hum. Nutr..

[23] El-Mostafa K., El Kharrassi Y., Badreddine A., Andreoletti P., Vamecq J., El Kebbaj M.S., Latruffe N., Lizard G., Nasser B., Cherkaoui-Malki M. (2014). Nopal cactus (*Opuntia ficus-indica*) as a source of bioactive compounds for Nutr.ition, health and disease. Molecules.

[24] Nassar A. (2008). Chemical composition and functional properties of prickly pear (*Opuntia ficus indica*) seeds flour and protein concentrate. World J. Dairy Food Sci..

[25] Piga A. (2004). Cactus pear: A fruit of Nutraceutical and functional importance. J. Prof. Assoc. Cactus.

[26] Ramadan M.F., Mörsel J.T. (2003). Oil cactus pear (*Opuntia ficus-indica* L.). Food Chem..

[27] StIntzing F.C., Schieber A., Carle R. (2001). Phytochemical and Nutr.itional significance of cactus pear. Eur. Food Res. Technol..

[28] Uchoa A.F., Souza P.A., Zarate R.M., Gomes-Filho E., Campos F.A. (1998). Isolation and characterization of a reserve protein from the seeds of *Opuntia ficus-indica* (Cactaceae). Braz. J. Med. Biol. Res..

[29] Kuti J.O. (2004). Antioxidant compounds from four Opuntia cactus pear fruit varieties. Food Chem..

[30] Impellizzeri G., Piattelli M. (1972). Biosynthesis of indicaxanthin in *Opuntia ficus-indica* fruits. Phytochemistry.

[31] StIntzing F.C., Schieber A., Carle R. (2002). Identification of betalains from yellow beet (*Beta vulgaris* L.) and cactus pear [*Opuntia ficus-indica* (L.) Mill.] by high-performance liquid chromatography-electrospray ionization mass spectrometry. J. Agric. Food Chem..

[32] Garzón G.A., Wrolstad R.E. (2001). The stability of pelargonidin-based anthocyanins at varying water activity. Food Chem..

[33] Tokgöz H., Gölükcü M., Toker R. (2013). Kan portakalı suyunun bazı kalite parametreleri üzerine ışık, pH, depolama, sıcaklık ve süresinin etkisi. Gıda Teknol. Elektron. Derg..

[34] Galati E.M., Tripodo M.M., Trovato A., Miceli N., Monforte M.T. (2002). Biological effect of *Opuntia ficus indica* (L.) Mill. (Cactaceae) waste matter. Note I: Diuretic activity. J. Ethnopharmacol..

[35] Loro J.F., del Rio I., Perez-Santana L. (1999). Preliminary studies of analgesic and anti-inflammatory properties of *Opuntia dillenii* aqueous extract. J. Ethnopharmacol..

[36] Park E.H., Kahng J.H., Lee S.H., Shin K.H. (2001). An anti-inflammatory principle from cactus. Fitoterapia.

[37] Galati E.M., Pergolizzi S., Miceli N., Monforte M.T., Tripodo M.M. (2002). Study on the increment of the production of gastric mucus in rats treated with *Opuntia ficus indica* (L.) Mill. cladodes. J. Ethnopharmacol..

[38] Lee E.B., Hyun J.E., Li D.W., Moon Y.I. (2002). Effects of *Opuntia ficus-indica* var. *saboten* stem on gastric damages in rats. Arch. Pharm. Res..

[39] Cardenas Medellin M.L., Serna Saldivar S.O., Velazco de la Garza J. (1998). Effect of raw and cooked nopal (*Opuntia ficus indica*) ingestion on growth and profile of total cholesterol, lipoproteins, and blood glucose in rats. Arch. Latinoam. Nutr..

[40] Frati A.C., Jimenez E., Ariza C.R. (1990). Hypoglycemic effect of *Opuntia-ficus-indica* in non-insulin-dependent diabetes-mellitus patients. Phytother. Res..

[41] Cao X., Wei Y., Ito Y. (2009). Preparative isolation of isorhamnetin from Stigma Maydis using high-speed countercurrent chromatography. J. Liq. Chromatogr. Relat. Technol..

[42] Ilhan M., Ali Z., Khan I.A., Tastan H., Kupeli Akkol E. (2019). Bioactivity-guided isolation of flavonoids from *Urtica dioica* L. and their effect on endometriosis rat model. J. Ethnopharmacol..

[43] Moniruzzaman M., Atikur Rahman M., Ferdous A. (2015). Evaluation of sedative and hypnotic activity of ethanolic extract of *Scoparia dulcis* Linn. Evid. Based Complement. Altern. Med..

[44] Takeda H., Tsuji M., Matsumiya T. (1998). Changes in head-dipping behavior in the hole-board test reflect the anxiogenic and/or anxiolytic state in mice. Eur. J. Pharmacol..

[45] Viola H., Wasowski C., Destein M.L., Wolfman C., Silveira R., Dajas F., Medina J.H., Paladini A.C. (1995). Apigenin, a component of *Matricaria recutita* flowers, is a central benzodiazepine receptors-ligand with anxiolytic effects. Planta Med..

[46] Muhammad N., Saeed M., Khan H., Haq I. (2013). Evaluation of *n*-hexane extract of *Viola betonicifolia* for its neuropharmacological properties. J. Nat. Med..

[47] Carvajal C.C., Vercauteren F., Dumont Y., Michalkiewicz M., Quirion R. (2004). Aged neuropeptide Y transgenic rats are resistant to acute stress but maInt.ain spatial and non-spatial learning. Behav. Brain Res..

[48] Li X.L., Aou S., Hori T., Oomura Y. (2002). Spatial memory deficit and emotional abnormality in OLETF rats. Physiol. Behav..

[49] Moragrega I., Carrasco M.C., Vicens P., Redolat R. (2003). Spatial learning in male mice with different levels of aggressiveness: Effects of housing conditions and nicotine administration. Behav. Brain Res..

[50] Rimondini R., Ågren G., Börjesson S., Sommer W., Heilig M. (2003). Persistent behavioral and autonomic supersensitivity to stress following prenatal stress exposure in rats. Behav. Brain Res..

[51] Alcalay R.N., Giladi E., Pick C.G., Gozes I. (2004). Intranasal administration of NAP, a neuroprotective peptide, decreases anxiety-like behavior in aging mice in the elevated plus maze. Neurosci. Lett..

[52] Ferguson G.D., Herschman H.R., Storm D.R. (2004). Reduced anxiety and depression-like behavior in synaptotagmin IV (−/−) mice. Neuropharmacology.

[53] Rodgers R.J., Cao B.J., Dalvi A., Holmes A. (1997). Animal models of anxiety: An ethological perspective. Braz. J. Med. Biol. Res..

[54] Prut L., Belzung C. (2003). The open field as a paradigm to measure the effects of drugs on anxiety-like behaviors: A review. Eur. J. Pharmacol..

[55] Candland D.K., Nagy Z.M. (1969). The open field: Some comparative data. Ann. N. Y. Acad. Sci..

[56] Lieben C.K.J., van Oorsouw K., Deutz N.E.P., Blokland A. (2004). Acute tryptophan depletion induced by a gelatin-based mixture impairs object memory but not affective behavior and spatial learning in the rat. Behav. Brain Res..

[57] Islam N.U., Khan I., Rauf A., Muhammad N., Shahid M., Shah M.R. (2015). Antinociceptive, muscle relaxant and sedative activities of gold nanoparticles generated by methanolic extract of *Euphorbia milii*. BMC Complement. Altern. Med..

[58] Sivam S.P., Nabeshima T., Ho I.K. (1982). Acute and chronic effects of pentobarbital in relation to postsynaptic GABA receptors: A study with muSci.mol. J. Neurosci. Res..

[59] Steinbach J.H., Akk G. (2001). Modulation of GABAA receptor channel gating by pentobarbital. J. Physiol..

[60] Awad R., Ahmed F., Bourbonnais-Spear N., Mullally M., Ta C.A., Tang A., Merali Z., Maquin P., Caal F., Cal V. (2009). Ethnopharmacology of Q’eqchi’ Maya antiepileptic and anxiolytic plants: Effects on the GABAergic system. J. Ethnopharmacol..

[61] Estrada-Reyes R., Lopez-Rubalcava C., Rocha L., Heinze G., Gonzalez Esquinca A.R., Martinez-Vazquez M. (2010). Anxiolytic-like and sedative actions of *Rollinia mucosa*: Possible involvement of the GABA/benzodiazepine receptor complex. Pharm. Biol..

[62] Fernandez S., Wasowski C., Paladini A.C., Marder M. (2004). Sedative and sleep-enhancing properties of linarin, a flavonoid-isolated from *Valeriana officinalis*. Pharmacol. Biochem. Behav..

[63] Kahnberg P., Lager E., Rosenberg C., Schougaard J., Camet L., Sterner O., Ostergaard Nielsen E., Nielsen M., Liljefors T. (2002). Refinement and evaluation of a pharmacophore model for flavone derivatives binding to the benzodiazepine site of the GABA(A) receptor. J. Med. Chem..

[64] Trofimiuk E., Walesiuk A., Braszko J.J. (2005). St John’s wort (*Hypericum perforatum*) diminishes cognitive impairment caused by the chronic restraint stress in rats. Pharmacol. Res..

[65] Rakhshandah H., Hosseini M., Doulati K. (2004). Hypnotic effect of *Rosa damascena* in mice. Iran. J. Pharm. Res..

[66] Girish C., Raj V., Arya J., Balakrishnan S. (2013). Involvement of the GABAergic system in the anxiolytic-like effect of the flavonoid ellagic acid in mice. Eur. J. Pharmacol..

[67] Medina J.H., Viola H., Wolfman C., Marder M., Wasowski C., Calvo D., Paladini A.C. (1997). Overview—Flavonoids: A new family of benzodiazepine receptor ligands. Neurochem. Res..

[68] Noguerón-Merino M., Jiménez-Ferrer E., Román-Ramos R., Zamilpa A., Tortoriello J., Herrera-Ruiz M. (2015). Interactions of a standardized flavonoid fraction from *Tilia americana* with serotoninergic drugs in elevated plus maze. J. Ethnopharmacol..

[69] Shephard R.A. (1987). Behavioral effects of GABA agonists in relation to anxiety and benzodiazepine action. Life Sci..

[70] Aguirre-Hernández E., González-Trujano M.E., Terrazas T., Santoyo J.H., Guevara-Fefer P. (2016). Anxiolytic and sedative-like effects of flavonoids from *Tilia americana* var. *mexicana*: GABAergic and serotonergic participation. Salud Ment..

[71] Priprem A., Watanatorn J., Sutthiparinyanont S., Phachonpai W., Muchimapura S. (2008). Anxiety and cognitive effects of quercetin liposomes in rats. Nanomedicine.

[72] Salgueiro J.B., Ardenghi P., Dias M., Ferreira M.B.C., Izquierdo I., Medina J.H. (1997). Anxiolytic natural and synthetic flavonoid ligands of the central benzodiazepine receptor have no effect on memory tasks in rats. Pharmacol. Biochem. Behav..

[73] Rice-Evans C., Miller N., Paganga G. (1997). Antioxidant properties of phenolic compounds. Trends Plant Sci..

[74] Dok-Go H., Lee K.H., Kim H.J., Lee E.H., Lee J., Song Y.S., Lee Y.H., Jin C., Lee Y.S., Cho J. (2003). Neuroprotective effects of antioxidative flavonoids, quercetin, (+)-dihydroquercetin and quercetin 3-methyl ether, isolated from *Opuntia ficus-indica* var. *saboten*. Brain Res..

[75] Ha H.J., Kwon Y.S., Park S.M., Shin T., Park J.H., Kim H.C., Kwon M.S., Wie M.B. (2003). Quercetin attenuates oxygen–glucose deprivation-and excitotoxin-induced neurotoxicity in primary cortical cell cultures. Biol. Pharm. Bull..

[76] Clarke S.E.D., Ramsay R.R. (2011). Dietary inhibitors of monoamine oxidase A. J. Neural. Transm..

[77] Asha D., Sumathi T. (2015). Isorhamnetin (IRN) attenuates cognitive dysfunction induced by the Int.racerebroventricular injection of amyloid beta 25–35 (Aβ 25–35) in Sprague Dawley rats. J. Pharm. Sci. Res..

[78] Li R., Guo M., Zhang G., Xu X., Li Q. (2006). Nicotiflorin reduces cerebral ischemic damage and upregulates endothelial nitric oxide synthase in primarily cultured rat cerebral blood vessel endothelial cells. J. Ethnopharmacol..

[79] Nakayama M., Aihara M., Chen Y.N., Araie M., Tomita-Yokotani K., Iwashina T. (2011). Neuroprotective effects of flavonoids on hypoxia-, glutamate-, and oxidative stress-induced retinal ganglion cell death. Mol. Vis..

[80] Huang J.L., Fu S.T., Jiang Y.Y., Cao Y.B., Guo M.L., Wang Y., Xu Z. (2007). Protective effects of nicotiflorin on reducing memory dysfunction, energy metabolism failure and oxidative stress in multi-infarct dementia model rats. Pharmacol. Biochem. Behav..

[81] Courvoisier S., Ducrot R., Julou L. (1957). Psychotropic Drugs.

[82] Laroche M.J., Rousselet F. (1990). Les Animaux de Laboratoire: Éthique et Bonnes Pratiques.

[83] Hoffmann G. (1963). Les Animaux de Laboratoire: Précis.

[84] Clark G., Koester A.G., Pearson D.W. (1971). Exploratory behavior in chronic disulfoton poisoning in mice. Psychopharmacologia.

[85] File S.E., Wardill A.G. (1975). Validity of head-dipping as a measure of exploration in a modified hole-board. Psychopharmacologia.

[86] Emamghoreishi M., Khasaki M., Aazam M.F. (2005). *Coriandrum sativum*: Evaluation of its anxiolytic effect in the elevated plus-maze. J. Ethnopharmacol..

[87] Consolini A.E., Ragone M.I., Migliori G.N., Conforti P., Volonte M.G. (2006). Cardiotonic and sedative effects of *Cecropia pachystachya* Mart. (ambay) on isolated rat hearts and conscious mice. J. Ethnopharmacol..

[88] Aziz A., Khan I.A. (2013). Pharmacological evaluation of sedative and hypnotic activities of methanolic extract of *Lycopus europaeus* in mice. J. Phytopharm..

[89] Herrera-Ruiz M., Gutierrez C., Enrique Jimenez-Ferrer J., Tortoriello J., Miron G., Leon I. (2007). Central nervous system depressant activity of an ethyl acetate extract from *Ipomoea stans* roots. J. Ethnopharmacol..

[90] Williamson E.M., Okpako D.T., Evans F.J. (1996). Selection, Preparation and Pharmacological Evaluation of Plant Material.

