# Supplementary material for: Sedative and Anxiolytic Activities of Opuntia ficus indica (L.) Mill.: An Experimental Assessment in Mice
Source: Molecules. 2020 Apr 16;25(8):1844. doi: 10.3390/molecules25081844 (PMC7221559; doi:10.3390/molecules25081844)
Supplement: Supplementary file 1 [file molecules-25-01844-s001.pdf]

^{1}H and ^{13}C-NMR data of compound **1, 2, 3,** and **4**

**C/H**	**1**		**2**		**3**		**4**	
	**δ$_H$ (mult., int., *J* (Hz))**	**δ$_C$**	**δ$_H$ (mult., int., *J* (Hz))**	**δ$_C$**	**δ$_H$ (mult., int., *J* (Hz))**	**δ$_C$**	**δ$_H$ (mult., int., *J* (Hz))**	**δ$_C$**
**2**	-	158.8	-	158.7	-	158.5	-	158.6
**3**	-	134.9	-	135.2	-	135.0	-	135.3
**4**	-	179.1	-	179.2	-	179.4	-	179.3
**5**	-	163.2	-	163.1	-	163.0	-	163.1
**6**	6.16 (bs, 1H)	100.1	6.17 (bs, 1H)	100.0	6.18 (bs, 1H)	100.3	6.18 (bs, 1H)	100.1
**7**		165.9		166.0		166.0	-	166.2
**8**	6.32 (bs, 1H)	94.7	6.37 (bs, 1H)	94.9	6.37 (bs, 1H)	94.9	6.37 (bs, 1H)	95.0
**9**	-	158.5	-	158.5	-	158.7	-	159.0
**10**	-	105.8	-	105.8	-	105.7	-	105.6
**1'**	-	123.0	-	123.1	-	123.0	-	122.8
**2'**	7.95 (d, 1H, 2.0 Hz)	114.1	7.95 (d, 1H, 2.0 Hz)	114.3	7.92 (d, 1H, 2.0 Hz)	114.2	8.05 (d, 1H, 8.6 Hz)	132.3
**3'**	-	148.2	-	148.3	-	148.1	6.89 (d, 1H, 8.6 Hz)	116.1
**4'**	-	150.6	-	150.8	-	151.0	-	161.5
**5'**	6.86 (d, 1H, 8.3 Hz)	115.9	6.87 (d, 1H, 8.5 Hz)	116.0	6.89 (d, 1H, 8.5 Hz)	116.2	6.89 (d, 1H, 8.6 Hz)	116.1
**6'**	7.57 (dd, 1H, 8.3/2.0 Hz)	123.4	7.58 (dd, 1H, 8.5/2.0 Hz)	123.8	7.57 (dd, 1H, 8.5/2.0 Hz)	123.5	8.05 (d, 1H, 8.6 Hz)	132.3
**3'-OCH_3**	3.91 (s, 3H)	56.2	3.93 (s, 3H)	56.3	3.94 (s, 3H)	56.6	-	-
**1''**			5.42 (d, 1H, 7.3 Hz)	103.6	5.40 (d, 1H, 7.3 Hz)	102.8	5.24 (d, 1H, 7.2 Hz)	104.1
**2''**				75.7		75.9		75.9
**3''**				78.4		78.3		78.5
**4''**			3.78-3.17	71.3	3.78-3.17	71.2	3.78-3.17	71.5
**5''**				78.0		77.9		78.0
**6''**				62.5		62.3		62.6
**1'''**					4.52 (bs, 1H)	102.3	4.52 (bs, 1H)	102.4
**2'''**						72.0		72.2
**3'''**					3.84-3.22	72.1	3.84-3.22	72.0
**4'''**						73.7		73.8
**5'''**						69.5		69.7
**6'''**					1.10 (d, 3H, 6.2 Hz)	17.4	1.13 (d, 3H, 6.2 Hz)	17.9

Mult.: multiplicity of the NMR signal; int: integral of the NMR signal (showing the number of H); J: coupling constant; bs: broad singlet; d: doublet
